# White matter-based brain network topological properties associated with individual impulsivity

**DOI:** 10.1038/s41598-023-49168-0

**Published:** 2023-12-13

**Authors:** Wi Hoon Jung, Euitae Kim

**Affiliations:** 1 Department of Psychology, Gachon University, 1342 Seongnam-daero, Sujeong-gu, Seongnam-si, Gyeonggi-do 13120 South Korea; 2 Department of Psychiatry, Seoul National University College of Medicine, Seoul, South Korea; 3 Department of Brain and Cognitive Sciences, Seoul National University College of Natural Sciences, Seoul, South Korea

**Keywords:** Emotion, Motivation, Social neuroscience, Neuroscience, Cognitive neuroscience, Decision, Psychiatric disorders

## Abstract

Delay discounting (DD), a parameter derived from the intertemporal choice task, is a representative behavioral indicator of choice impulsivity. Previous research reported not only an association between DD and impulsive control disorders and negative health outcomes but also the neural correlates of DD. However, to date, there are few studies investigating the structural brain network topologies associated with individual differences in DD and whether self-reported measures (BIS-11) of impulsivity associated with DD share the same or distinct neural mechanisms is still unclear. To address these issues, here, we combined graph theoretical analysis with diffusion tensor imaging to investigate the associations between DD and the topological properties of the structural connectivity network and BIS-11 scores. Results revealed that people with a steep DD (greater impatience) had decreased small-worldness (a shift toward weaker small-worldnization) and increased degree centrality in the medial superior prefrontal cortex, associated with subjective value in the task. Though DD was associated with the BIS-11 motor impulsiveness subscale, this subscale was linked to topological properties different from DD; that is, high motor impulsiveness was associated with decreased local efficiency (less segregation) and decreased degree centrality in the precentral gyrus, involved in motor control. These findings provide insights into the systemic brain characteristics underlying individual differences in impulsivity and potential neural markers which could predict susceptibility to impulsive behaviors.

## Introduction

Impulsivity is considered both an aspect of personality and a prominent feature of various psychiatry disorders and abnormal behaviors, including attention-deficit/hyperactivity disorder (ADHD), addiction, and aggression^1^. Researchers suggest that impulsivity is a complex, multifactorial concept defined as a tendency to act without forethought, an inability to inhibit inappropriate behaviors, as well as intolerance of delay gratification (i.e., choosing short-term rewards over long-term ones, referred to as impulsive choice or reward-delay impulsivity)^2–4^. Accordingly, various measures including self-report personality questionnaires and behavioral tasks have been developed to assess impulsivity and each of its separate components, which represent different underlying processes^5,6^.

One widely used behavioral indicator of impulsivity (particularly for choice impulsivity) is delay discounting (DD), the tendency to which individuals discount the outcome value based on the time to their receipt. An individual’s DD (quantified as a discount rate *k*) can be measured using a history of choices between small-but-immediate and large-but-delayed rewards in an intertemporal choice task (also called DD task)^6^. In other words, DD values can be quantified by applying specific approaches to behavioral data obtained during the task^7^. For example, DD is quantified by estimating the *k* parameter according to a specific theoretical model (e.g., exponential model^8^ or hyperbolic^9^ model used in this study). Atheoretical methods can also be adopted to estimate DD (e.g., area under the indifference curve, AUC^10^). In many previous studies, an association between DD (calculated as mentioned above) and disorders of impulsive control (such as ADHD and substance use disorder)^11,12^ and negative health outcomes in real life has been described (e.g., obesity and texting while driving)^13,14^. Therefore, investigating the neural mechanisms underlying DD can provide not only important clues about individual differences in DD but also potential neural markers to predict susceptibility to impulsive and addictive behaviors. Accordingly, various functional neuroimaging studies on DD have found DD-associated specific brain regions, including those encoding the subjective value of given options during the task (ventral striatum, medial prefrontal cortex, and posterior cingulate cortex)^15–17^; regions associated with cognitive control (lateral prefrontal cortex and lateral parietal cortex)^16–21^; and regions associated with imagining prospective events (medial temporal lobe and anterior temporal cortex)^22,23^. In addition, neuroanatomical studies using diffusion tensor imaging (DTI) have observed that DD is associated with structural connectivity in frontostriatal white matter and lateral prefrontal cortex tracts^24–26^.

Previous studies have often reported an association between DD and self-reported measures of impulsivity^27,28^. For example, DD is associated with some Barratt Impulsiveness Scale (BIS-11) scores^27,29^. The BIS-11, one of the most common used self-report measures, is the gold standard for assessing trait impulsivity with three components: attentional (lack of focus on a task at hand), motor (acting without thinking), and non-planning impulsiveness (lack of regard for the future)^30^. However, significant associations between these two measures (DD and BIS-11) of impulsivity are not consistently reported^31^, showing either an association of DD with total^29^ or certain BIS-11 subscale scores^28,32,33^; or no association at all (neither with total nor BIS-11 subscales)^34–36^. Therefore, the identity of most related aspects of trait impulsivity from BIS-11 to DD remains unclear. In addition, it is unclear whether DD and its associated BIS-11 subscales are based on the same or distinct neural correlates. In this regard, identifying common and distinct neural correlates between these measures could provide valuable insights into the nature of the brain systems underlying impulsive behaviors as well as therapeutic targets for the treatment of maladaptive impulsivity (e.g., targets for neuromodulation by transcranial magnetic stimulation (TMS) and transcranial direct current stimulation (tDCS)^37^).

Graph theoretical analysis (GTA) is used to investigate large-scale complex brain networks^38,39^. Particularly, GTA of DTI data can quantify the topological characteristics of a structural brain network constructed from region-to-region white matter connectivity^39,40^. An important global topological property of brain networks is its small-worldness^38^. The small-worldness metric reflects a balance between segregation and integration of information processing in the network, as it is calculated by comparing the clustering coefficient (which indicates the extent of local interconnectivity in the network as a measure of information segregation) and characteristic path length (which indicates the average length over all shortest paths in the network as a measure of information integration) with those of matched random networks. Global efficiency and local efficiency are other global measures of information integration and segregation, respectively^41,42^. Global efficiency is inversely related to the characteristic path length as the measure of efficiency in information transmission among all pairs of nodes in the network, while the local efficiency is related to the clustering coefficient as the measure of efficiency in information transmission limited to neighboring nodes^43^.

Local topological properties in the network can be also captured by nodal characteristics including degree centrality [number of nodes (i.e., brain regions) connected to the current node in a network] and betweenness centrality (number of times a node stands on the shortest path between other nodes in a network). These nodal metrics are used to determine important nodes as network hubs^44^. Previous GTA studies of DTI structural networks have revealed associations between network measures derived from GTA and cognitive function (e.g., intelligence quotient)^45^ and mental health symptoms^46,47^. However, to date, few studies have investigated associations between DD and the topological properties of structural and functional brain networks using DTI and resting-state fMRI data, showing that high discounters had reduced small-worldness^48^. In addition, to our knowledge, no research has explicitly compared the neural mechanisms underlying impulsivity assessed by DD and the BIS-11 (as behavioral and self-reported measures of impulsivity, respectively) using GTA.

Thus, we aimed to clarify whether the individual DD (i.e., the *k* parameter estimated by hyperbolic model;^9^) would be associated with global and local topological properties of a DTI-based structural network. To this end, we measured both the global network metrics (representing information integration [characteristic path length and global efficiency], information segregation [clustering coefficient and local efficiency], and the balance between them [small-worldness]) and local network metrics (including degree centrality and betweenness centrality). Based on previous research^48,49^, we hypothesized that the structural network underlying higher impulsivity would have lower global topological features, such as lower small-worldness. In addition, we investigated whether DD was associated with particular BIS-11 subscales, and whether these subscales share neural mechanisms in terms of the topological properties of the DTI-based structural network.

## Materials and methods

### Participants

Participants were part of the Psychological and Neural Mechanisms for Predicting Academic Achievement (PNMPAA) study. For the PNMPAA study, participants performed several behavioral tasks, including a modified incentive delay task, an intertemporal choice task, and a risk tolerance task, and underwent brain scans. The scanning session consisted of high-resolution T1-weighted anatomical Magnetic Resonance Imaging (MRI), resting-state fMRI, DTI, and fMRI during cognitive tasks. A previous report described the involvement of the posterior parietal cortex on regulatory focus (a motivational construct in goal pursuit^50^) using data from the modified incentive delay task. In the present study, we used behavioral data obtained from the tasks performed outside the MRI scanner before the scan.

All participants in the present study were young healthy adults who provided written informed consent before participation. All study procedures were approved by the Institutional Review Board of Gachon University. All methods were conducted in accordance with the relevant guidelines and regulations.

Of all data (n = 115) collected to date, 73 participants completed both the intertemporal choice task, BIS-11 questionnaire, and brain scans. Four individuals out of the 73 were excluded due to missing DTI data. Another four participants were excluded for extreme decision preferences on the task performance (people who chose only the same one of two options across all trials). Therefore, 65 participants (37/28 male/female; age [mean ± standard deviation (SD)], 22.062 ± 2.766 years; duration of education, 15.046 ± 1.304 years; DD, 0.0164 ± 0.0137) were used in the final analysis.

### Intertemporal choice task

**Figure 1:**
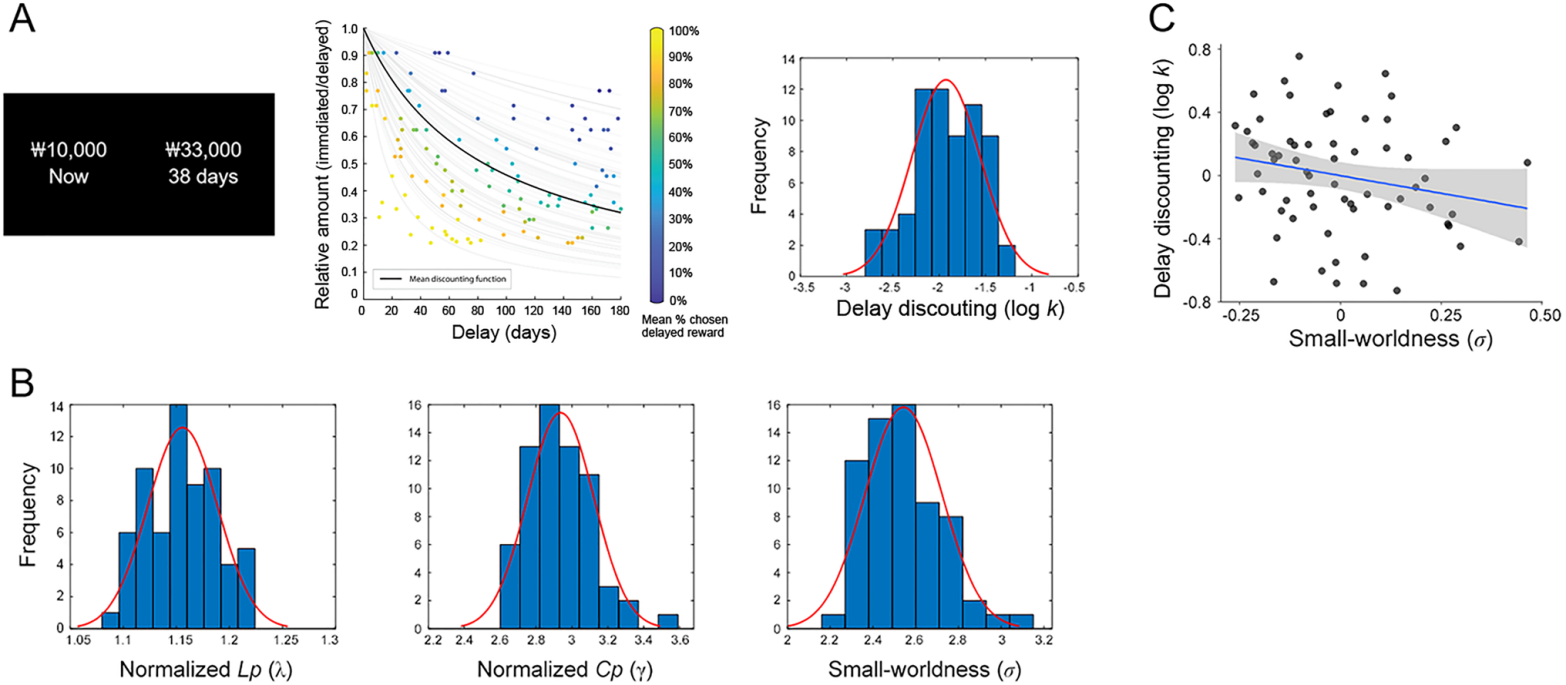
Distribution of individuals’ delay discounting (DD) rate and global topological measures of the structural network and their associations. (**A**) Sample trials (left panel) in the intertemporal choice task, hyperbolic curve fitting to behavioral data (middle panel), and distribution of individual DD values (right panel). Left panel: Participants were asked to make a choice between a smaller-immediate reward and a larger-delayed reward. Middle panel: Individuals’ DD rates were estimated from behavioral data during the task. We plotted each trial as a function of the delay on the abscissa and the relative amount, which is obtained by dividing the immediate amount by the delayed amount, on the ordinate. In addition to the two axes, we color-coded each trial by the proportion of participants who chose the delayed option and overlaid a hyperbolic function based on the mean discount rate *k* (black) across all participants, as well as all the individually fitted discount functions (gray). (**B**) Distribution of global topological properties of the structural brain network. For all participants, the structural network showed small-world architecture ($\Upsilon$ > 1, $\lambda \approx 1$, and *σ* > 1). (**C**) Association between DD and small-worldness. Higher DD was associated with lower small-worldness. For illustration purposes, this partial correlation scatterplot was generated by performing Pearson correlation analysis between residuals after regressing out age, sex, and education years.

The task was performed outside the MRI scanner prior to the scan. Participants were asked to make a series of 120 choices between a smaller-immediate reward, fixed at ₩10,000 (roughly $8–$9 USD) for all trials and a variable larger-delayed reward (Fig. 1A). The magnitude of the larger-delayed reward ranged from ₩11,000 to ₩48,000, and the delay could be 2–180 days. Individual behavioral data were fit with a logistic regression function using maximum likelihood estimation to capture the probability of choosing the larger-delayed reward as a stochastic function of the difference in subjective value (*SV*) between the two options. Based on standard behavioral findings^6,9^, we assumed that *SV* is a hyperbolic function of the reward amount (*A*) and delay (*D*): *SV* = *A*/(1 + *kD*), where *k* is the participant’s discount rate. Larger *k* values represent a greater degree of discounting future rewards. The *k* (referred to as DD in this manuscript) was log-transformed to normalize the distribution before statistical analyses.

### Barratt impulsiveness scale-11

The BIS-11 is composed of 30 items rated with a 4-point Likert scale from 1 (rarely/never) to 4 (almost always/always)^30^. The items are categorized into three subscales: attentional impulsiveness (attention and cognitive instability), motor impulsiveness (motor and perseverance), and non-planning impulsiveness (self-control and cognitive complexity). Higher scores represent greater impulsivity. All participants completed the BIS-11 (total score [mean ± SD], 49.708 ± 8.921; attentional impulsiveness, 14.031 ± 3.250; motor impulsiveness, 15.200 ± 4.032; non-planning impulsiveness, 20.477 ± 4.135). In this study, Cronbach’s alpha for the internal consistency reliability was 0.82.

### Image acquisition

All imaging data were acquired on a 3 T Trio MRI scanner (Siemens, Erlangen, Germany). High-resolution T1-weighted anatomical images were obtained using a 3D magnetization-prepared rapid-gradient echo (MPRAGE) sequence (repetition time [TR] = 1,900 ms, echo time [TE] = 2.52 ms, flip angle [FA] = 9°, voxel size = 1.0 × 1.0 × 1.0 mm^3^, 192 sagittal slices). DTI data were acquired using a single-shot multiband echo-planar imaging sequence (TR = 3,000 ms, TE = 70 ms, FA = 90°, multiband acceleration factor = 3, phase partial Fourier = 6/8, voxel size = 2.0 × 2.0 × 2.0 mm^3^, 75 interleaved axial slices, and 64 diffusion directions with b-values of 1,000 s/mm^2^ and eight images with b-values of 0 s/mm^2^).

### Image preprocessing and network construction

Data were preprocessing using PANDA v1.3.1^51^ (https://www.nitrc.org/projects/panda/): a pipeline software for diffusion MRI that uses functions of FSL (https://fsl.fmrib.ox.ac.uk/fsl/fslwiki/), Pipeline System for Octave and Matlab (PSOM), and the Diffusion Toolkit (https://www.nitrc.org/projects/trackvis/). Eddy current correction was applied to correct distortions and head movements. The average image of b0 images was used as reference to register the diffusion-weighted images. Whole-brain fiber tracking was conducted using the Fiber Assignment by Continuous Tracking (FACT) algorithm^52,53^ with the fractional anisotropy threshold set at 0.20 and the tracking turning angular threshold set at 45°. Afterwards, spline filtering was applied to smooth the streamline tractography.

The 90 regions (45 per hemisphere) of the cerebrum from the Anatomical Automatic Labeling (AAL) atlas^54^ were used as network nodes. Then, the network matrix with edges (i.e., structural connectivity) between 90 nodes for each participant was estimated in the native diffusion space^44,48^; T1-weighted MPRAGE images were co-registered to the native space of the b0 images through linear transformation. Then, the co-registered T1 images were nonlinearly registered to the Montreal Neurological Institute (MNI)-152 T1-template space. The obtained parameters were applied to warp the AAL template to the native diffusion space. Consequently, the connecting fiber number (FN) between two nodes (*i* and *j*), mean FA of the connecting fiber, and the average volume of the two nodes were calculated. Finally, we quantified the weights of the edges between two nodes by multiplying FN by the mean FA along the fibers connecting a pair of regions, normalized by dividing the average volume of the two connecting regions to reduce the bias, where larger cortical regions may receive more virtual fibers (*W*_*ij*_ = FN × FA/volume)^53,55,56^.

### Graph theoretical-based network analysis

GTA was performed using the Graph Theoretical Network Analysis (GRETNA) toolbox (https://www.nitrc.org/projects/gretna)^57^. Global and local (nodal) network topological properties were estimated for the weighted structural network matrix derived from each participant. The global measures included clustering coefficient (*Cp)*, characteristic path length (*Lp)*, small-worldness (*σ*), global efficiency (*E*_*glob*_), and local efficiency (*E*_*loc*_). The local topological measures included degree centrality and betweenness centrality.

The equations used to calculate these measures can be found elsewhere^41,42^. Briefly, *Cp* is defined as the average of the likelihood of a neighbor-to-neighbor connection over all nodes in a network; a greater value indicates a larger extent of local interconnectivity in a network^58^. *Lp* is defined as the average of all shortest path lengths between each pair of nodes in the network; a smaller value indicates a faster information transfer in the network^59^. To examine the small-world properties, the *Cp* and *Lp* values were normalized in comparison with those of 100º-matched random networks $(\Upsilon= {C}_{P}^{real}/{C}_{P}^{rand} and \lambda = {L}_{P}^{real}/{L}_{P}^{rand}; \sigma = \Upsilon/ \lambda )$^60^. Typically, a small-world network should meet the following criteria: $\Upsilon$ > 1, $\lambda \approx 1$, and *σ* > 1^58,61^. *E*_*glob*_ is defined by the inverse of the average harmonic of the shortest path length between each pair of nodes^62^. The *E*_*loc*_ of a node *i* represents the fault tolerance level of the network when the first neighbors of a node *i* are removed from the network^63^. The global *E*_*loc*_ is defined as the average of the nodal *E*_*loc*_ values in the network. Degree centrality is defined as the connection of a node *i* with all other nodes in the network. Betweenness centrality is defined as the fraction of all the shortest paths in the network running through a node *i*^64^. Before statistical analyses, the degree centrality and betweenness centrality of a node *i* were normalized by dividing their average values of the network, respectively. Hubs were defined as regions with values > 1 SD above the average of all nodes in the network.

### Statistical analysis

To test whether individual differences in DD are significantly associated with either the structural network topological properties or BIS-11 scores, we performed Spearman’s rank correlation between the log-transformed DD rate and each global and local topological measure (and each BIS-11 score), after regressing out covariate effects (age, sex, and education years). In case that any BIS-11 scores were associated with individual DD rates, we further tested whether the score was associated with any network topological properties to clarify whether DD rates and BIS-11 scores are related to the same or distinct topological properties. The level of significance of all statistical tests was set at p < 0.05 based on previous GTA studies^42,48,65–69^. For local topological properties, we corrected the significance for the number of nodes using a less stringent false positive adjustment (i.e., *p* < (1/90) = 0.011) as reported in previous studies^69–71^.

## Results

### Global network properties associated with DD

The whole-brain structural network showed a small-world architecture ($\Upsilon$ > 1, $\lambda \approx 1$, and *σ* > 1; Fig. 1B) for all participants ($\Upsilon$ [mean ± SD], 2.937 ± 0.185; $\lambda$, 1.156 ± 0.033; *σ,* 2.543 ± 0.180).

The individual DD was negatively correlated with small-worldness (*σ*, r-/p-values =  − 0.252/0.043), indicating that higher DD was associated with less small-worldness (Fig. 1C). Other global properties were not associated with DD ($\Upsilon$, r-/*p*-values = 0.140/0.267; $\lambda$, − 02.40/0.055; *E*_*glob*_, 0.113/0.369; *E*_*loc*_, − 0.078/0.539).

### Local network properties associated with DD

**Figure 2:**
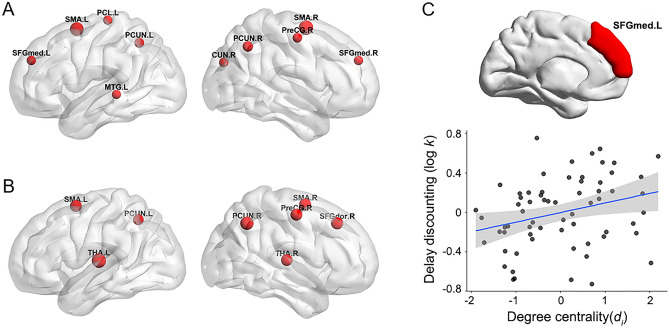
Local topological measures of the structural network and DD. (**A**) Hub regions identified from degree centrality values. (**B**) Hub regions identified from betweenness centrality values. Most hubs were located in frontal and parietal areas. (**C**) Association between DD and the degree centrality of the left medial superior frontal gyrus; that is, a higher DD was associated with a greater degree centrality of the left medial superior frontal gyrus, associated with SV in the intertemporal choice task. For illustration purposes, this partial correlation scatterplot was generated by performing Pearson correlation analysis between residuals after regressing out age, sex, and education years. L, left; R, right; SFGmed, medial superior frontal gyrus; SMA, supplementary mother area; PCL, paracentral lobule; PCUN, precuneus; MTG, middle temporal gyrus; CUN, cuneus; THA, thalamus; SFGdor, dorsal superior frontal gyrus.

The identified hub regions were the right precentral gyrus, bilateral supplementary motor area, and bilateral precuneus based on degree centrality (*d*_*i*_) and betweenness centrality (*b*_*i*_) values (Fig. 2). The bilateral medial superior frontal gyrus (SFGmed), right cuneus, left paracentral lobule, and left middle temporal gyrus were also considered hub regions based on degree centrality (Fig. 2A) whereas the left dorsal superior frontal gyrus and bilateral thalamus were added based on betweenness centrality (Fig. 2B).

Based on the significance threshold we set, individual DD correlated only with the degree centrality of the left SFGmed (r-/*p*-values = 0.330/0.008), a region identified as a hub (Fig. 2C). Other nodal centrality values showed no significant correlations with DD (all *p* > 0.011). The regions with the highest correlation coefficients (top 10%) between DD and degree centrality or betweenness centrality values are shown in Supplementary Information.

### Association with BIS-11

**Figure 3:**
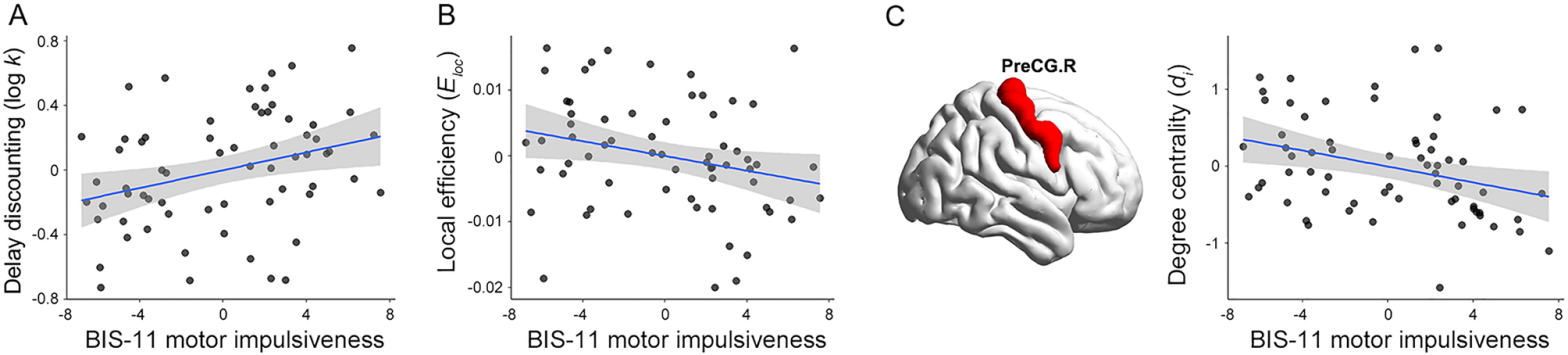
Global and local topological measures of the structural network and BIS-11 motor impulsiveness subscale. (**A**) Positive association between DD and motor impulsiveness subscale score. (**B**) Association between motor impulsiveness subscale score and local efficiency; that is, higher motor impulsiveness was associated with lower local efficiency. (**C**) Association between motor impulsiveness subscale score and the degree centrality of the right precentral gyrus; that is, higher motor impulsiveness was associated with less degree centrality of the right precentral gyrus, involved in controlling motor movement. For illustration purposes, these scatterplots were generated by performing Pearson correlation analysis between residuals after regressing out age, sex, and education years.

Among BIS-11 total and subscale scores, individual DD was positively correlated only with the motor impulsiveness subscale (r-/*p*-value = 0.322/0.009; Fig. 3A), indicating that greater DD was associated with higher motor impulsivity. Other BIS-11 scores were not correlated with DD (attentional impulsiveness, r-/*p*-values = -0.018/0.886; non-planning impulsiveness, 0.205/0.102; total score, 0.219/0.080).

The motor impulsiveness score was negatively correlated with local efficiency (*E*_*loc*_; r-/*p*-*values* =  − 0.280/0.024; Fig. 3B) and the degree centrality of the right precentral gyrus (r-/*p*-values =  − 0.384/0.002; Fig. 3C), indicating that higher motor impulsivity correlates less with these two topological properties. There were no significant correlations between the motor impulsiveness score and global (*σ*, r-/p-values =  − 0.146/0.247; $\Upsilon$, − 0.110/0.383; $\lambda$, − 0.227/0.069; *E*_*glob*_, = 0.156/0.213; *arz*, − 0.044/0.726) and local topological properties (all ps > 0.011). The regions with the highest correlation coefficients (top 10%) between motor impulsivity and degree centrality or betweenness centrality values are presented in Supplementary Information.

## Discussion

In this study, we aimed to determine whether individual DD (a representative behavioral indicator of choice impulsivity) estimated from the intertemporal choice task, is associated with the topological properties of the large-scale structural brain network constructed based on region-to-region white matter connectivity using GTA. We found an association between DD and certain global and local topological properties of the structural brain network. Among the global topological properties, a higher DD (i.e., steeper DD) was associated with less small-worldness. In addition, DD had the strongest positive association with the degree centrality in SFGmed, a region known to encode SV. We then tested the association between individual DD and total and subscale scores of the BIS-11 (the gold standard, self-reported measure of impulsivity) and found a significant positive relationship between individual DD and the motor impulsiveness subscale score. We further investigated whether DD and its associated BIS-11 subscale (motor impulsiveness subscale score) are linked to the same or distinct topological properties of the structural brain network. Contrary to findings with DD, the motor impulsiveness subscale score was associated with local efficiency and the degree centrality of the precentral gyrus, a region associated with motor control. These results suggest that these two associated measures are related to different network topological properties. Collectively, our findings suggest that global and local topological properties of the DTI-based structural brain network underline individual differences in impulsivities measured by the intertemporal choice task (DD) and BIS-11 self-report questionaries (BIS-11 motor impulsiveness subscale score) but they (i.e., the DD and BIS-11) are associated with different topological properties.

A small-world network is considered the most efficient model allowing efficient communication over both short and long distances, as it is characterized by a high extent of local interconnectivity and short path length between nodes (i.e., brain regions) in a network^58^. In the present study, all DTI-based structural brain networks from each participant exhibited a small-world architecture. We also found that individual DD was negatively associated with small-worldness, which indicates that people with higher DD (greater impatience) had lower small-worldness (less balance between segregation and integration in the structural brain network), consistent with previous findings in healthy young adults^48^. Therefore, based on our current and previous findings, it appears that efficient communication throughout all brain regions (an optimal balance between network segregation and integration) is important in controlling impulsiveness, particularly impulsive choice. Indeed, previous studies in clinical patients who lacked impulsive control showed abnormal small-worldness in functional and structural brain networks^72–75^.

Hubs are important regions crucial for efficient communication in a network^76^. Most identified hubs were located in frontal and parietal areas, regions which functionally belong to association areas, except for the precentral gyrus (primary area) and the thalamus (subcortical area). Association areas are high-order multimodal parts of the cerebral cortex that receive inputs from diverse areas to integrate information from various areas of the brain^77^. In the present study, individual DD was positively associated with the degree centrality of the left SFGmed. That is, people with greater impatience had higher degree centrality in the left SFGmed. A previous meta-analysis of fMRI studies on reward processing showed that the SFGmed is involved in both positive and negative effects of SV on neural activity, suggesting that this region is associated with signals of arousal or salience^78^. Moreover, recent studies have demonstrated that the SFGmed (i.e., dorsomedial prefrontal cortex) encodes SV across the self and other in the intertemporal and risky choice tasks^79,80^. Given that the degree centrality of a node indicates its number of connections^41,42^ and that, in the present study, the SFGmed was a hub in the network, the SFGmed might have more connections from various areas than other areas. Thus, impatience may be related to aberrations in structural white matter connectivity (e.g., redundant fiber bundles and reduced prefrontal pruning)^81,82^ in this region, associated with SV in the intertemporal choice task.

In the present findings individual DD was positively associated with the motor impulsiveness (acting without thinking) subscale scores of BIS-11. Albeit inconsistently, associations between DD and BIS-11 as a representative behavioral and self-report measure of impulsivity respectively have been repeatedly reported. For instances, some studies have reported the association of DD with attentional^28,32^, motor^83^, non-planning impulsiveness subscale scores^28,33^ or total scores of BIS-11 while others have reported no association between DD and BIS-11 (total nor subscales) scores^34–36^. One explanation proposed by de Wit et al.^33^ for these discrepancies is that self-report measures of impulsivity (i.e., BIS-11) reflect participants’ perceptions of their behaviors across various situations and times whereas the intertemporal choice task for DD measures a specific behavior (choices between two options) at a specific time point in an experimental context. Another explanation could be differences in sample characteristics; that is, participants’ demographic and personal characteristics may affect their responses. For example, individual differences in DD have been associated with age^84,85^, intelligence quotient^33^, and extraversion^86^. In addition, some studies have reported the impact of affective state on the association between DD and BIS-11, especially with respect to the motor impulsiveness subscale^83,87^. McLeish and Oxoby^83^ observed that the results on the motor impulsiveness subscale were associated with DD only after receiving negative feedback. Moreover, Koff and Lucas^87^ reported that individuals with high negative affect showed a stronger relationship between DD and the motor impulsiveness subscale than those with low negative affect and, based on the negative urgency (the tendency to behave rashly in response to negative affect mentioned by Whiteside and Lynam^88^), they suggested that individuals may engage in impulsive behaviors to decrease negative emotions; in other words, an individual may choose more immediate rewards under negative affective states. Therefore, further research is needed to clarify whether there of the affective state has an influence on the association between DD and the BIS-11 subscale we observed as well as their neural correlates.

In the present study, while individual DD was negatively associated with small-worldness among global topological measures, the BIS-11 motor impulsiveness subscale, associated with DD, was negatively linked to local efficiency. In other words, people with higher motor impulsiveness had lower local efficiency. Given that the local efficiency of a network is defined as the average of local efficiencies (the efficiency of information transfer from a node to its neighboring ones) of all nodes and that it indicates the degree of segregation in the network^41,63^, people with higher motor impulsiveness may have weaker communication between close areas. Regarding nodal topological measures, while individual DD was positively associated with the degree centrality of the left SFGmed, motor impulsiveness was negatively associated with the degree centrality of the right precentral gyrus. Given that the precentral gyrus, referred to as the primary motor cortex, is responsible for the control of motor movement, people with high motor impulsiveness may have aberrant structural connections with regions involved in response inhibition. For instance, Hampton et al.^26^ found that motor impulsivity (as measured by go/no-go task) was associated with individual differences in structural connectivity between dorsal striatum and motor areas of response inhibition, including supplementary motor area. Future research should aim to clarify the disruption of structural connectivity associated with response inhibition in people with motor impulsivity.

It is interesting to note the involvement of SFGmed and precentral gyrus in choice impulsivity and motor impulsivity, respectively. Because this would strengthen the hypothesis that medial and lateral prefrontal areas are crucial sites for what are called “hot executive functions (EFs)” and “cold EFs”, respectively^89^. Choice impulsivity and motor impulsivity are each types of impulsivity, but belong to different EFs. Hot EFs are involved in processing information derived from the context of emotion, reward, and motivation (e.g., DD and emotion regulation) and mainly engage the medial and orbitofrontal cortex (OFC). Cold EFs, on the other hand, involve purely cognitive information processing (e.g., response inhibition and working memory) and are mainly associated with the lateral prefrontal cortex including dorsolateral prefrontal cortex (DLPFC) and ventrolateral prefrontal cortex^89,90^. In the present study, the DLPFC and OFC, which are involved in the DD^91,92^, were not included in the hubs identified through GTA. Considering that the present study used the structural connectivity network, the hub would be an area with many direct connections (i.e., fibers) between brain regions. Therefore, areas involved in organizing information that comes from various other areas (associated with complex functions including hot and cold EFs and sensory and motor information, e.g., association areas) may act as hubs.

Many previous studies have investigated the effect of neuromodulation on impulsivity by TMS and tDCS^37,93^. Especially, the vast majority of neuromodulation studies in DD have targeted the DLPFC, OFC, ventromedial prefrontal cortex, and temporoparietal junction, but the results of these studies are inconsistent (e.g., either increases or decreases in DD^37,92^). The regions (the left SFGmed and right precentral gyrus) identified in the present study may be proposed as potential targets for neuromodulation of impulsivity. Considering that these identified regions were based on the degree centrality of white matter connectivity (i.e., the fibers directly connecting brain regions), it is expected that stimulating these regions can have a direct effect on several regions connected to them. Future research is needed to validate this notion.

Several limitations in the present study should be mentioned. First, this study included only young, healthy adults. It is thus necessary to corroborate this results in people with impulsive disorders or older people (given the effect of age on DD). Second, given that structural connectivity networks are considered as the physical substrate of functional connectivity networks, our explanations for the present findings are mostly based on previous functional research. Future studies need to uncover the role of the SFGmed and precentral gyrus on DD and their functional connection with other brain regions using fMRI data. Finally, based on many previous studies^48,65–69^, an uncorrected p value of 0.05 was used to examine the association of global topological features with DD. This threshold has been widely adopted as the empirical significance level for statistical models in many GTA studies^48,65–69^, though the results do not guarantee against false positives. Additionally, to date, few studies have investigated the association between whole-brain organization patterns (i.e., whole-brain network topological features) and DD. Therefore, to comprehensively explore whole-brain network topological features relevant to DD, the liberal statistical threshold may provide more information about the association between them compared to overly conservative multiple comparison corrections.

## Conclusions

In summary, the present study examined the neural mechanisms underlying individual differences in DD (a behavioral indicator of choice impulsivity) and further investigated whether a DD-associated self-report measure of impulsivity (the BIS-11 subscale) would be based on common or distinct neural mechanisms, as shown by the global and local topological properties of a white matter-based structural connectivity network determined using DTI. Our findings showed an association between DD and small-worldness and the degree centrality of the SFGmed, suggesting the importance of a balance between information segregation and integration and the connection with the SFGmed, a region which encodes SV, in impulsive choice. Our findings also showed an association between DD and the BIS-11 motor impulsiveness subscale but the neural mechanisms differed between these two impulsivity measures. In addition, the motor impulsiveness subscale was associated with local efficiency and the degree centrality of the precentral gyrus, suggesting a role in information segregation and motor control. These findings provide insights into the brain characteristics underlying individual differences in impulsivity and suggest potential therapeutic targets for the treatment of impulsivity.

### Supplementary Information

Supplementary Tables.

## Data Availability

The datasets generated and analyzed for the present study are available from the corresponding author on reasonable request.
