# Supplementary material for: White matter-based brain network topological properties associated with individual impulsivity
Source: Sci Rep. 2023 Dec 13;13:22173. doi: 10.1038/s41598-023-49168-0 (PMC10719274; doi:10.1038/s41598-023-49168-0)
Supplement: Supplementary file 1 — Supplementary Tables. [file 41598_2023_49168_MOESM1_ESM.pdf]

Supplementary Table 1. Regions with the highest correlation coefficients (top 10%) between delay discounting rate and degree centrality or betweenness centrality

#Rank	Area	Correlation with *DC*		Area	Correlation with *BC*	
		r-value	p-value		r-value	p-value
1	SFGmed.L	0.330	0.008*	ROL.R	0.287	0.020
2	SMG.L	0.313	0.011	MFG.R	-0.280	0.024
3	STG.L	-0.309	0.013	MTG.L	-0.257	0.038
4	PreCG.L	-0.305	0.014	SFGmed.L	0.226	0.070
5	PoCG.L	0.303	0.015	PreCG.L	-0.226	0.070
6	ROL.R	0.294	0.018	IPL.R	-0.226	0.071
7	SFGmed.R	0.258	0.039	PCG.L	0.215	0.086
8	ANG.L	0.238	0.057	STG.L	-0.214	0.086
9	IPL.L	0.232	0.063	CAU.R	-0.214	0.086

DC, degree centrality; *BC*, betweenness centrality; L, left; R, right; SFGmed, medial superior frontal gyrus; SMG, supramarginal gyrus; STG, superior temporal gyrus; PreCG, precentral gyrus; PoCG, postcentral gyrus; ROL, rolandic operculum; ANG, angular gyrus, IPL, inferior parietal lobule; MFG, middle frontal gyrus; PCG, posterior cingulate gyrus; CAU, caudate nucleus.* $p < 0.011$

Supplementary Table 2. Regions with the highest correlation coefficients (top 10%) between BIS-11 motor impulsiveness score and degree centrality or betweenness centrality

#Rank	Area	Correlation with *DC*		Area	Correlation with *BC*	
		r-value	p-value		r-value	p-value
1	PreCG.R	-0.384	0.002*	TPOsup.L	0.261	0.036
2	IPL.R	-0.286	0.021	CAL.L	-0.250	0.045
3	SMA.L	-0.282	0.023	PCUN.L	-0.205	0.101
4	IOG.R	-0.268	0.031	ANG.L	0.204	0.103
5	SPG.R	-0.246	0.048	IPL.R	-0.201	0.109
6	MFG.L	0.241	0.053	ANG.R	0.188	0.133
7	TPOsup.L	0.237	0.057	IOG.R	-0.184	0.142
8	PCUN.L	-0.229	0.067	DCG.L	0.181	0.148
9	REC.L	-0.187	0.137	REC.L	-0.180	0.151

DC, degree centrality; *BC*, betweenness centrality; L, left; R, right; PreCG, precentral gyrus; IPL, inferior parietal lobule; SMA, supplementary motor area; IOG, inferior occipital gyrus; SPG, superior parietal gyrus; MFG, middle frontal gyrus; TPOsup, superior temporal pole; PCUN, precuneus; REC, gyrus rectus; CAL, calcarine; ANG, angular gyrus; DCG, median cingulate and paracingulate gyri. * $p < 0.011$
